# The Effect of Single and Multiple SERAT Mutants on Serine and Sulfur Metabolism

**DOI:** 10.3389/fpls.2018.00702

**Published:** 2018-05-28

**Authors:** Mutsumi Watanabe, Takayuki Tohge, Alisdair R. Fernie, Rainer Hoefgen

**Affiliations:** ^1^Max Planck Institute of Molecular Plant Physiology, Potsdam, Germany; ^2^Nara Institute of Science and Technology, Ikoma, Japan

**Keywords:** serine acetyltransferase, serine, *O*-acetylserine, cysteine, subcellular compartment

## Abstract

The gene family of serine acetyltransferases (SERATs) constitutes an interface between the plant pathways of serine and sulfur metabolism. SERATs provide the activated precursor, *O*-acetylserine for the fixation of reduced sulfur into cysteine by exchanging the serine hydroxyl moiety by a sulfhydryl moiety, and subsequently all organic compounds containing reduced sulfur moieties. We investigate here, how manipulation of the SERAT interface results in metabolic alterations upstream or downstream of this boundary and the extent to which the five SERAT isoforms exert an effect on the coupled system, respectively. Serine is synthesized through three distinct pathways while cysteine biosynthesis is distributed over the three compartments cytosol, mitochondria, and plastids. As the respective mutants are viable, all necessary metabolites can obviously cross various membrane systems to compensate what would otherwise constitute a lethal failure in cysteine biosynthesis. Furthermore, given that cysteine serves as precursor for multiple pathways, cysteine biosynthesis is highly regulated at both, the enzyme and the expression level. In this study, metabolite profiles of a mutant series of the *SERAT* gene family displayed that levels of the downstream metabolites in sulfur metabolism were affected in correlation with the reduction levels of SERAT activities and the growth phenotypes, while levels of the upstream metabolites in serine metabolism were unchanged in the *serat* mutants compared to wild-type plants. These results suggest that despite of the fact that the two metabolic pathways are directly connected, there seems to be no causal link in metabolic alterations. This might be caused by the difference of their pool sizes or the tight regulation by homeostatic mechanisms that control the metabolite concentration in plant cells. Additionally, growth conditions exerted an influence on metabolic compositions.

## Introduction

Serine is an amino acid that plays an indispensable role in plant metabolism, development and cell signaling. Importantly, serine is essential as building block of proteins and as precursor of many biomolecules that have diverse biological roles including nucleic acids, lipids and amino acids such as cysteine and tryptophan (Tzin and Galili, 2010; Ros et al., 2014). In plants, cysteine is produced from an activated serine, *O*-acetylserine (OAS) in a two-step reaction sequence catalyzed by two enzymes; (i) serine acetyltransferase (SERAT) which catalyzes the formation of OAS from serine and acetyl coenzyme A and (ii) *O*-acetylserine(thiol)lyase (OASTL) which catalyzes the formation of cysteine from OAS and sulfide produced by sulfate reduction. SERAT thus forms an interface between serine and cysteine (sulfur-) metabolism. Cysteine is used for the synthesis of another sulfur-containing amino acid, methionine, and many sulfur-containing metabolites including cofactors, vitamins, glutathione (GSH), and glucosinolates (GSLs) which are secondary metabolites specific for plants of the *Brassicales* clade (Hesse and Hoefgen, 2003; Takahashi et al., 2011; Koprivova and Kopriva, 2016). Since cysteine biosynthesis is a key branch-point between serine metabolism as nitrogen/carbon source and the sulfur assimilation pathway, the function and regulation of both, SERAT and OASTL, have been studied intensively with assessing their subcellular localizations (reviewed in Hell and Wirtz, 2011).

In contrast to the fact that the reduction steps of sulfate to sulfide are localized mainly in plastids, serine, OAS and cysteine are synthesized in multiple subcellular compartments in plants (Hell and Wirtz, 2011; Ros et al., 2014). Serine biosynthesis occurs via three different pathways (reviewed in Ros et al., 2014); (i) the glycolate/photorespiratory pathway via glycine in mitochondria, (ii) the phosphorylated pathway via phosphorylated metabolites from 3-phosphoglycerate in the plastids and (iii) the glycerate pathway in the cytosol or peroxisomes. Results obtained by combination of enzymatic assays, metabolite profiling and gene expression analyses using Arabidopsis T-DNA knockout mutants and transgenic overexpression lines of the genes involved in the serine metabolism suggested that the photorespiratory pathway is the main serine source in photosynthetic tissues (Voll et al., 2006; Engel et al., 2011) while the phosphorylated pathway contributes to serine production in the dark, in non-photosynthetic tissues or in specific cells (Ho et al., 1999; Ho and Saito, 2001; Pagnussat et al., 2005; Benstein et al., 2013; Cascales-Miñana et al., 2013; Toujani et al., 2013; Ros et al., 2014). However, the significance and contribution of the glycerate pathway has not been studied well yet. Similar studies focusing on the sulfur metabolism suggested that OAS and cysteine are synthesized in three compartments, namely cytosol, plastids, and mitochondria with multiple isoforms of SERATs and OASTLs. In Arabidopsis, five SERATs (SERAT1;1, SERAT2;1, SERAT2;2, SERAT3;1, and SERAT3;2) (**Table 1**) and three OASTLs (OASTL1;1, OASTL2;1, and OASTL2;2) were identified, and their contributions to OAS and cysteine synthesis are different depending on plant tissues and growth conditions (Wirtz and Hell, 2007; Haas et al., 2008; Heeg et al., 2008; Watanabe et al., 2008a,b; Krueger et al., 2009; Birke et al., 2012). Interestingly, in the case of Arabidopsis, mitochondrial SERAT2;2 was mainly responsible for OAS formation in photosynthetic leaf tissues, while serine formation was also dominant in the mitochondria (Haas et al., 2008; Watanabe et al., 2008b; Krueger et al., 2009), suggesting that there are specific subcellular metabolic networks in plants, which should be analyzed to understand metabolic regulations which maintain the balance of carbon/nitrogen/sulfur in each subcellular compartment.

**Table 1:** *SERAT* gene family.

Gene name	AGI code	Localization
SERAT1;1	At5g56760	Cytosol
SERAT2;1	At1g55920	Plastids
SERAT2;2	At3g13110	Mitochondria
SERAT3;1	At2g17640	Cytosol
SERAT3;2	At4g45640	Cytosol

Despite of the fact that OAS and cysteine are synthesized in multiple subcellular compartments, Arabidopsis knockout mutants lacking one or retaining only one compartment specific SERAT or OASTL isoform, respectively, did not provoke any lethal phenotype although a quintuple mutant, where all five SERATs were knocked out, did (Watanabe et al., 2008a,b). This experimental evidence indicates that OAS or cysteine can be exchanged between compartments to compensate for the loss of OAS and cysteine biosynthesis in the mutants irrespective of the subcellular compartment. In contrast, the Arabidopsis knockout mutants of serine metabolism defective in the photorespiratory pathway in mitochondria showed a severe photorespiratory phenotype of chlorosis at ambient CO_2_ (Voll et al., 2006; Engel et al., 2011) and mutants defective in the glycolate pathway in plastids showed developmental defects in embryos, male gametophytes and roots (reviewed in Ros et al., 2014), which suggested that the transport of serine between compartments encounters some limitations or that the three serine pathways are strictly regulated with respect to timing and tissue specificity resulting in an inability to compensate for the loss of serine in either compartment.

Biosynthetic pathways of serine and OAS/cysteine have been well studied individually, however, the connection and the coordination of the three serine pathways and the OAS/cysteine pathways in each subcellular localization have as yet not been well addressed. In this study, a mutant series of the *SERAT* gene family lacking or retaining OAS production in only one of the subcellular compartments; *serat1;1, serat2;1, serat2;2, serat3;1, serat3;2* as single knockout mutants and the previously published quadruple mutants Q1;1 (*serat2;1serat2;2serat3;1serat3;2*), Q2;1 (*serat1;1serat2;2serat3;1serat3;2*), Q2;2 (*serat1;1serat2; 1serat3;1serat3;2*), Q3;1 (*serat1;1serat2;1serat2;2serat3;2*), Q3;2 (*serat1;1serat2;1serat2;2serat3;1*) (Watanabe et al., 2008b), were investigated as tools to study the link of serine biosynthetic pathways with OAS/cysteine biosynthetic pathways and the effects of manipulating this serine/cysteine interface on metabolism, both upstream and downstream by determining metabolite profiles.

## Results

### Growth Phenotypes of *serat* Mutants

**FIGURE 1:**
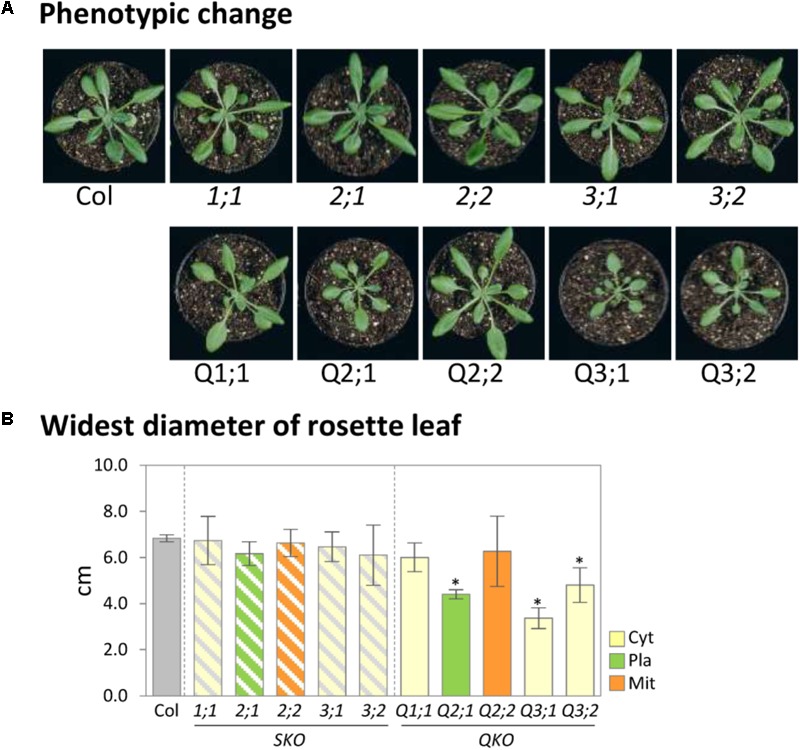
Growth phenotypes of *serat* mutants. **(A)** Phenotypic changes of *serat* mutants. Plants were grown for 25 days on soil in plant pots of 6 cm diameter. **(B)** Widest diameter of rosette leaves of 25-day-old plants. Plants were grown on soil. Data represent the mean (±SD) of three biological replicates. Differences between the wild type and *serat* mutants were analyzed using Student’s *t*-test and statistical significance was indicated (^∗^*P* < 0.05). Cyt, cytosol; Pla, plastids; Mit, mitochondria. SKO, *serat* single mutants (hatched bars); QKO, *serat* quadruple mutants (color bars).

We evaluated the growth phenotype of *serat* mutants under defined, more natural growth conditions (long-day, on soil; see “growth condition” in section “Materials and Methods”) in comparison to previous studies on seedlings grown on agar plates. Growth phenotypes of sulfur metabolism-related mutants were reported to be affected by growth conditions such as light or nutrient conditions (Wirtz and Hell, 2007; Haas et al., 2008; Heeg et al., 2008; Watanabe et al., 2008a,b; Krueger et al., 2009; Bermudez et al., 2010) since the expression levels of genes involved in sulfur metabolism were regulated by those conditions (Watanabe et al., 2010; Caldana et al., 2011). Under our growth conditions, growth retardation was significantly observed in the mutants Q2;1 with a decrease of the widest leaf diameter to 64.4% of the wild-type plant, Q3;1 (49.3%) and Q3;2 (70.2%) (**Figures 1A,B**). This result was similar to the growth phenotype of *serat* mutants grown on agar plates under long-day conditions (Watanabe et al., 2008b) with the exception that the Q3;2 mutant, which showed the most severe growth retardation on plates, grew better than the Q3;1 mutant under our soil based growth conditions.

### SERAT Activity in *serat* Mutants

**FIGURE 2:**
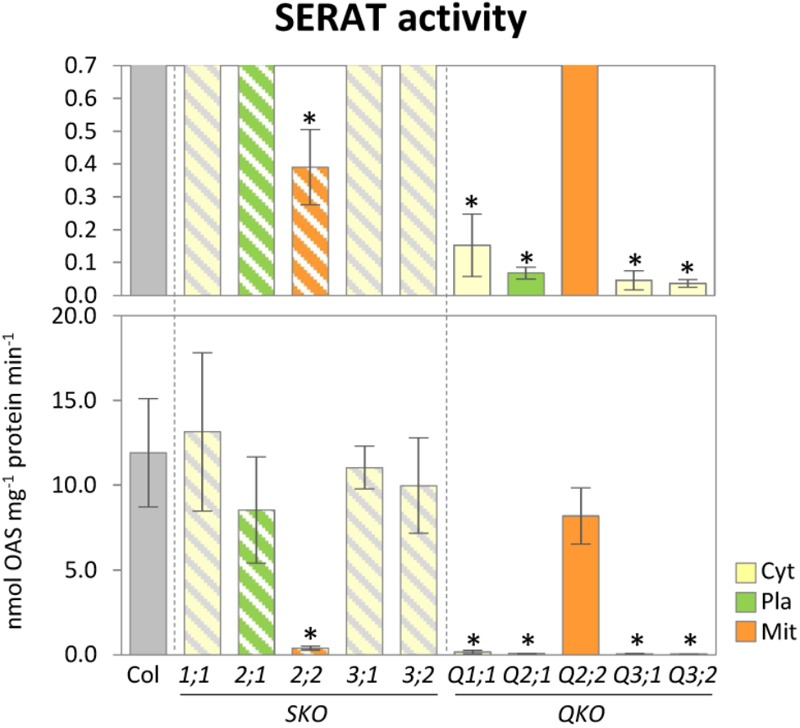
Enzymatic activities of SERAT in *serat* mutants. Enzymatic activities of SERAT. Enzymatic activities were determined using total extracts of soluble proteins prepared from rosette leaves of 25-day-old plants. Data represent the mean (±SD) of three biological replicates. Differences between the wild type and *serat* mutants were analyzed using Student’s *t*-test and statistical significance was indicated (^∗^*P* < 0.05). Cyt, cytosol; Pla, plastids; Mit, mitochondria. SKO, *serat* single mutants (hatched bars); QKO, *serat* quadruple mutants (color bars).

To investigate the contribution of individual SERAT isoforms on the total cellular activities, the SERAT activities in leaves were determined in crude protein extracts from rosette leaves of 25-day-old plants (**Figure 2**). Significant reductions in SERAT activity were observed in *serat2;2* (decreased to 3.3% of the wild-type plant) and the four quadruple mutants Q1;1 (1.3%), Q2;1 (0.6 %), Q3;1 (0.4%), and Q3;2 (0.3%), suggesting the predominant contribution of the mitochondrial SERAT2;2 to the total SERAT activity in leaves of Arabidopsis, which is consistent with previous studies (Haas et al., 2008; Watanabe et al., 2008b; Krueger et al., 2009).

### Metabolite Changes in Sulfur and Serine Metabolism in *serat* Mutants

**FIGURE 3:**
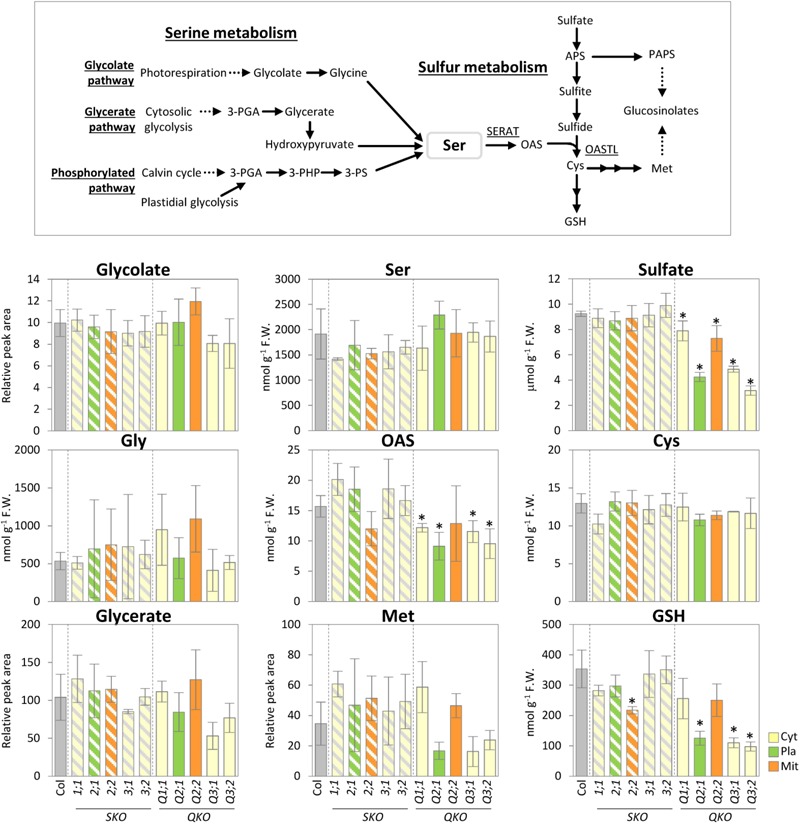
Accumulation of serine and sulfur metabolism in *serat* mutants. Schematic representation of serine and sulfur metabolism and changes of the selected metabolites in rosette leaves of 25-day-old plants. Contents of glycolate, glycerate, and Met were determined by Gas Chromatography/Time of Flight-Mass Spectrometer (GC/TOF-MS) analysis, contents of OAS, Ser and thiols (Cys and GSH) by HPLC analysis and sulfate by ion chromatography. In GC/TOF-MS analysis peak area was normalized to sample fresh weight and the peak area of internal standard. Data represent the mean (±SD) of three biological replicates. Differences between the wild type and *serat* mutants were analyzed using Student’s *t*-test and were marked when statistically significant (^∗^*P* < 0.05). Abbreviations: 3-PGA, 3-phosphoglycerate; 3-PHP, 3-phosphohydroxypyruvate; 3-PS, 3-phosphoserine; Ser, serine; OAS, *O*-acetyl serine; APS, 5′-adenosine phosphosulfate; PAPS, 3′-phosphoadenosine-5′-phosphosulfate; Cys, cysteine; GSH, glutathione; Met, methionine; SERAT, serine acetyltransferase; OASTL, *O*-acetylserine(thiol)lyase. Cyt, cytosol; Pla, plastids; Mit, mitochondria. SKO, *serat* single mutants (hatched bars); QKO, *serat* quadruple mutants (color bars).

To investigate to which extent the mutation of the various individual SERATs affects OAS production in each subcellular compartment and, hence, metabolite compositions in upstream (serine), and downstream (sulfur) metabolism, we performed metabolite analyses of rosette leaves of 25-day-old *serat* mutants (**Figure 3**). Levels of the product of SERAT, OAS, was significantly decreased in the quadruple mutants compared to wild-type plant with the exception of Q2;2. Levels of the subsequent metabolite, cysteine, and methionine were not significantly altered in any of the *serat* mutants, but a further downstream metabolite, GSH, was significantly decreased in *serat2;2* and the growth-retarded mutants Q2;1, Q3;1, and Q3;2. The initial metabolite of the sulfur assimilation pathway, namely sulfate, was significantly decreased in all the quadruple mutants. In contrast to the significant changes in sulfur metabolism correlating with the growth phenotypes and the reduction levels of SERAT activities, levels of the metabolites upstream of OAS in serine metabolism such as serine, glycine, glycolate, and glycerate were not significantly altered in the *serat* mutants compared to wild-type plants.

### Changes in Glucosinolate Compositions in *serat* Mutants

**FIGURE 4:**
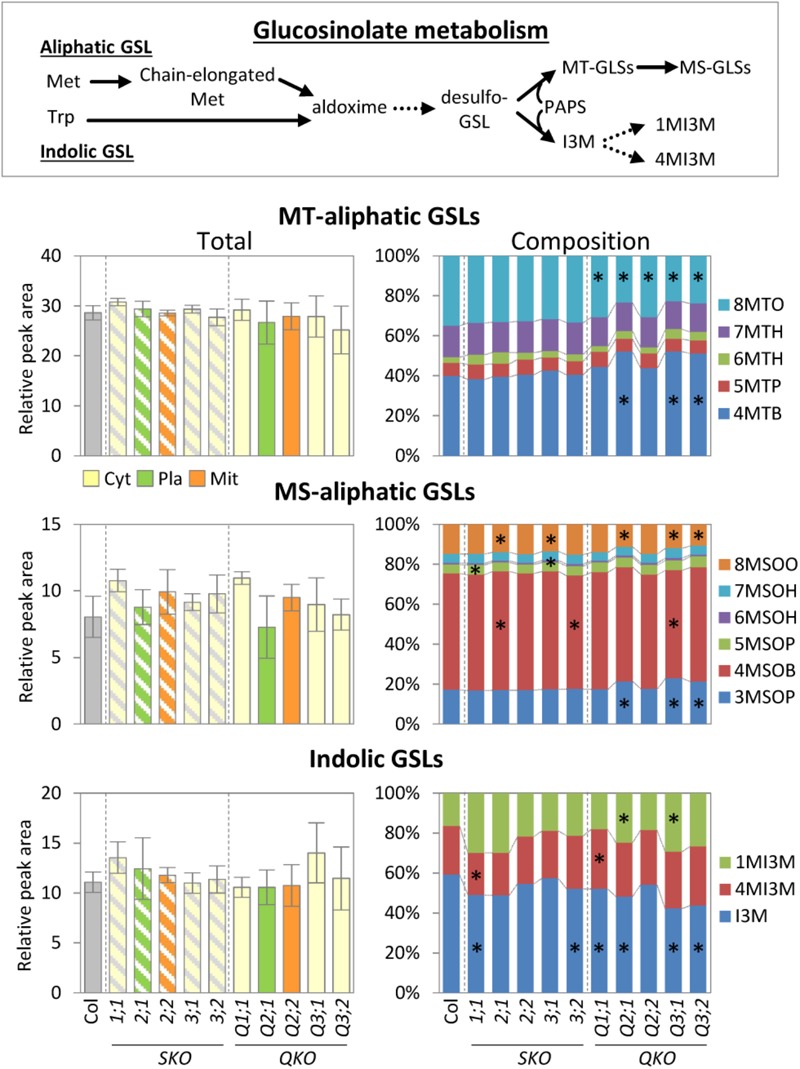
Accumulation of glucosinolate metabolism in *serat* mutants. Changes of total methylthioalkyl (MT-) aliphatic, methylsulfinylalkyl (MS-) aliphatic, indolic glucosinolate (GSL) contents in rosette leaves of 25-day-old plants. Contents of GSLs were determined by Liquid Chromatography/ElectroSpray Ionization-Mass Spectrometry (LC/ESI-MS) analysis. Peak area was normalized to sample fresh weight and the peak area of an internal standard. Data represent the mean (±SD) of three biological replicates. Percentages in stacked bar plots are displayed as mean percentage between three replicates. Differences between the wild type and *serat* mutants were analyzed using Student’s *t*-test and statistical significance was indicated (^∗^*P* < 0.05). Abbreviations: Met, methionine; Trp, tryptophan; PAPS, 3′-phosphoadenosine-5′-phosphosulfate; MT-aliphatic GLSs (4MTB, 5MTP, 6MTH, 7MTH, and 8MSOO, for 4-methylthiobutyl-GSL, 5-methylthiopentyl-GSL, 6-methylthiohexyl-GSL, 7-methylthioheptyl-GSL, and 8-methylthiooctyl-GSL, respectively); MS-aliphatic GLSs (3MSOP, 4MSOB, 5MSOP, 6MSOH, 7MSOH, and 8MSOO, for 3-methylsulfinylpropyl-GSL, 4-methylsulfinylbutyl-GSL, 5-methylsulfinylpentyl-GLS, 6-methylsulfinylhexyl-GLS, 7-methylsulfinylheptyl-GLS, and 8-methylsulfinyloctyl-GLS, respectively); indolic GLSs (I3M, 4MI3M, and 1MI3M, for indolyl-3-methyl-GSL, 4-methoxy-indolyl-3-methyl-GSL, and 1-methoxy-indolyl-3-methyl-GSL). Cyt, cytosol; Pla, plastids; Mit, mitochondria. SKO, *serat* single mutants (hatched bars); QKO, *serat* quadruple mutants (color bars).

Glucosinolates are sulfur-containing secondary metabolites found in Brassicaceae, such as Arabidopsis. GSLs play a variety of roles in plant defense (Bednarek et al., 2009) and are presumed to have a sulfur storage role (Schnug, 1990; Hirai et al., 2004, 2005; Falk et al., 2007). In Arabidopsis leaves the major types are methionine-derived aliphatic GSLs (methylsulfinylalkyl [MS]- and methylthioalkyl [MT]-aliphatic GSLs) with variations in the side-chain length and tryptophan-derived indole GSLs (Brown et al., 2003). The total GSL levels were not significantly changed in any of the *serat* mutants when compared to wild-type plants (**Figure 4**). However, the GSL compositions with respect to different chain lengths were significantly altered in *serat* mutants, particularly in the three growth-retarded mutants Q2;1, Q3;1, and Q3;2. The ratios of long chain MS- and MT-aliphatic GSLs (8-methylthiooctyl-GSL [8MTO] and 8-methylsulfinyloctyl-GSL [8MSOO]) in total MS- and total MT-aliphatic GSLs, respectively, were decreased whilst those of short chain aliphatic GSLs (4-methylthiobutyl-GSL [4MTB] and 3-methylsulfinylpropyl GSL [3MSOP]) were increased in Q2;1, Q3;1, and Q3;2 mutants. The remaining quadruple mutants Q1;1 and Q2;2 showed a significant reduction in the ratio of 8MTO. Single *serat* mutants showed some significant reductions of MS-aliphatic GSLs, but the differences were relatively small (0.9–1.1-fold change compared to wild-type plants). A change in composition of indolic GSLs was also observed in single and quadruple mutants. The growth-retarded quadruple mutants showed a trend for a decrease of the ratio of indolyl-3-methyl-GSL (I3M) and increase of the ratio of 4-methoxy-indolyl-3-methyl-GSL (1MI3M) although the change of 1MI3M in Q3;2 was not statistically significant.

### Metabolite Changes in Indirectly Related Metabolic Pathways in *serat* Mutants

**FIGURE 5:**
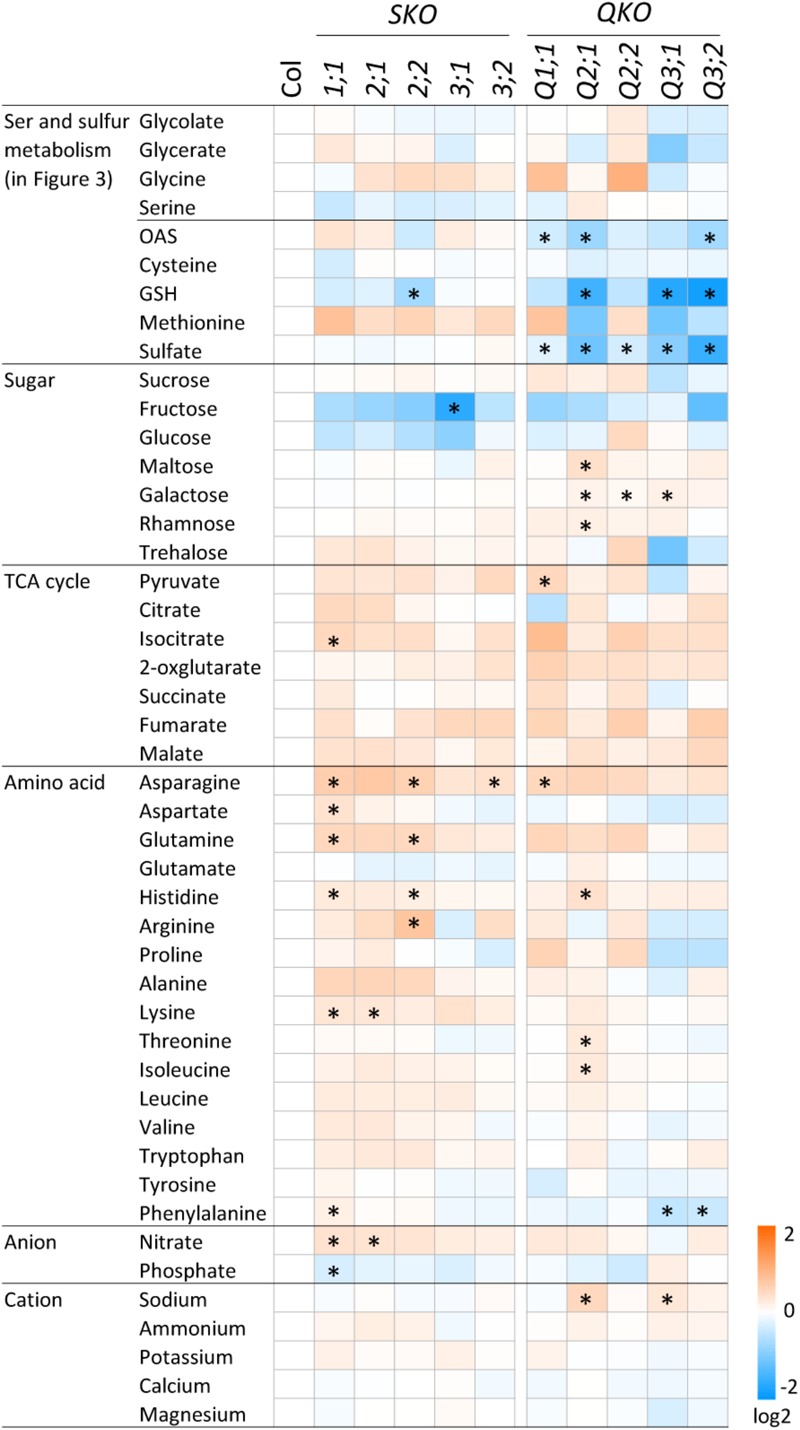
Accumulation of primary metabolites in *serat* mutants. Changes in primary metabolite contents in rosette leaves of 25-day-old plants. Contents of sugars, organic acids in the tricarboxylic acid (TCA) cycle and proline were determined by GC/TOF-MS. Peak area was normalized to sample fresh weight and the peak area of internal standard. Contents of amino acids were determined by HPLC analysis and ions by ion chromatography. Log2 ratios of fold changes from wild-type plant (Col-0) are given by shades of red or blue colors according to the scale bar. Data represent the mean (±SD) of three biological replicates. Differences between the wild type and *serat* mutants were analyzed using Student’s *t*-test and statistical significance was indicated (^∗^*P* < 0.05).

To investigate the metabolic changes caused by the perturbation of OAS production and the subsequent sulfur metabolism in the *serat* mutants, we analyzed contents of the sugars, organic acids of the tricarboxylic acid (TCA) cycle, amino acids, and ions (**Figure 5**). There were several metabolites with statistically significant differences in *serat* mutants compared to the wild-type plants, although the differences were generally relatively small (less than fold-changes of >1.5 or <0.66 for increase or decrease, respectively). That said, asparagine was increased in *serat1;1* (1.56-fold) and *serat2;2* (1.51-fold), arginine was increased in *serat2;2* (1.69-fold), and fructose was decreased in *serat3;1* (0.32-fold). Furthermore, there were a few metabolites showing a clear negative correlation between single and quadruple mutants or changing in a subcellular localization specific manner. The growth-retarded mutants, Q3;1 and Q3;2 showed similar metabolite changes with a significant reduction of phenylalanine and a slight decrease in some amino acids such as, aspartate, arginine, proline and a sugar, trehalose, most of which were also decreased in plants grown under sulfur depleted conditions (Nikiforova et al., 2005), suggesting that the metabolic responses of Q3;1 and Q3;2 partially mimic sulfate depletion responses.

## Discussion

### Relationship Between Serine Metabolism and OAS Production

*O*-acetylserine production by SERAT takes place independently in three compartments, the cytosol, the plastids, and most prominently within the mitochondria in Arabidopsis leaves (Haas et al., 2008; Watanabe et al., 2008b; Krueger et al., 2009). SERAT activity provides the only link between glycine/serine and cysteine/methionine metabolism. We therefore expected that manipulating OAS production in either of the respective subcellular compartments should affect the distribution or content of the OAS precursor serine in plant cells. In this study, however, the serine content was not changed in any of the *serat* mutants in a statistically safe manner (**Figure 3**). One explanation for this might be that the pool size of serine is about 100-fold bigger than those of OAS or cysteine, and even about fivefold higher than for GSH and, thus, there is only a marginal effect on the serine pool if SERAT activities are impaired. Serine content in wild-type plants in this study was 1914.0 nmol g^-1^ fresh weight (F.W.), whilst contents of OAS, cysteine and GSH were 15.7, 12.9, and 353.8 nmol g^-1^ F.W., respectively (**Figure 3**). In Arabidopsis leaves, production of serine and OAS occur mainly in mitochondria (Voll et al., 2006; Haas et al., 2008; Watanabe et al., 2008b; Krueger et al., 2009; Engel et al., 2011), suggesting high accumulation of serine and OAS in the mitochondria. It could thus be speculated that serine biosynthesis is not regulated by its downstream products such as OAS, cysteine, GSH or others, which change when altering SERAT expression. Significant changes only occur downstream of SERAT (**Figure 3**) which can be explained through the feedback regulatory properties of the sulfur assimilation and sulfur metabolism pathways (Takahashi et al., 2011). Thus, SERAT constitutes an interface at the border of two unlinked or barely linked metabolic areas despite their immediate biochemical linkage, at least under soil borne growth conditions. Another possibility is that serine biosynthesis is tightly regulated by the serine concentration itself through feedback inhibition mechanisms of the serine biosynthetic enzymes (Okamura and Hirai, 2017) or at the transcriptional levels of genes in serine biosynthesis (Timm et al., 2013) in order to keep serine concentrations constant irrespective of linked pathways.

### Metabolite Changes of *serat* Mutants Depend on Grown Conditions

The growth-retarded phenotypes following the reduction of SERAT activity in the quadruple mutants Q2;1, Q3;1, and Q3;2 (**Figures 1, 2**) were observed also in the mutants grown on full nutrient agar-plates containing 1% sucrose under long-day conditions (Watanabe et al., 2008b). However, the metabolite changes were quite different between the two conditions. Both OAS and GSH were decreased in the *serat* mutants in this study (**Figures 2, 3**), but were unchanged in Q2;1 and Q3;1 grown under the agar-plate condition although the contents were low in Q3;2 (Watanabe et al., 2008b, 2010). Despite the fact that there is no change of OAS and GSH, the Q2;1, Q3;1, and Q3;2 mutants remarkably accumulated some amino acids such as serine and glycine upstream of the SERAT interface, as well as glutamine (Watanabe et al., 2008b), which also accumulated in sulfur-starved plants (Nikiforova et al., 2005; Hoefgen and Nikiforova, 2008). In conclusion, when grown on agar plates the metabolic responses except for the sulfur metabolites in SERAT Q2;1, Q3;1, and Q3;2 mutants resembled those of sulfate starved plants. The gene expression analysis of the agar plate grown quadruple mutants with microarrays (Watanabe et al., 2010) further suggested that the accumulation of serine and glycine might be caused by the upregulation of several genes in the phosphorylated pathway for serine production and the downregulation of serine hydroxymethyltransferase1 (SHM1) as a major isoform in the photorespiratory pathway (Voll et al., 2006; Engel et al., 2011) for a decrease of conversion from glycine to serine. These results therefore suggested the existence of some regulatory connection between serine and sulfur metabolism, at least under certain specific conditions. Such upregulation of genes in the phosphorylated pathway and the downregulation of *SHM1* were also observed in senescent plants or in plants grown under several nutrient depletion conditions such as sulfate, nitrate, and phosphate depletions (Watanabe et al., 2010), suggesting that the expression of the genes of the serine pathways might rather be regulated by a general senescence program, not by a sulfur specific response. The precise mechanism underlying the remarkable metabolic and transcriptomic responses in the *serat* mutants remains unclear. The balance of supply and demand of serine for production of OAS and further downstream metabolites such as cysteine and methionine, which again act as precursors for other metabolic pathways, or GSH for protection against oxidative stress, which also could be modulated by several factors in the environment might be a key factor for determining whether the metabolic homeostasis could be maintained or not in plant cells. This idea is supported by the difference in change of OAS in *serat*2;2 mutant between this study (**Figure 3**) and previous study with agar-plate system (Watanabe et al., 2008b). In this study OAS in the *serat*2;2 mutant was not changed significantly in comparison with wild-type plant, but the downstream metabolite GSH was reduced while in the previous study OAS was reduced significantly, but GSH was not changed. It suggests that the steady state levels of metabolites in plant cells could be differently adjusted to the limited availability of OAS in the *serat*2;2 mutant depending on the environment. The differences of the metabolic changes in *serat* mutants depending on the growth conditions seem likely to be caused by the differences between the soil conditions in this study and the agar-plate conditions. Relevant factors might be, (i) the nutrition including the sucrose supply under the agar-plate conditions, which affects photosynthesis and photorespiration in plants (Zakhartsev et al., 2016), (ii) the light environment, especially as roots were in the dark under soil conditions, but exposed to light under the agar-plate conditions, which affects the whole metabolism in roots (Xu et al., 2013), and (iii) the plant age as 25-day-old plants were used under soil conditions and 14-day-old plants under the agar-plate conditions, which affects the photosynthetic performance (Bielczynski et al., 2017), metabolic process (Watanabe et al., 2013), and adaptation to stresses (Skirycz et al., 2010). In contrast to the differences of the metabolic changes depending on the growth conditions, the levels of SERAT activities in *serat* mutants in comparison to wild type were very similar in both growth conditions (the soil and the agar-plate) (**Figure 2**; Watanabe et al., 2008b), suggesting that the contribution of each SERAT isoform to the total SERAT activity is stable in Arabidopsis green leaf, independent of the respective growth conditions. This might be due to the specific gene expression levels (abundant) and patterns (tissue specificity) of the *SERAT*s. Among the five SERAT isoforms, SERAT1;1, 2;1 and 2;2 were biochemically the dominant forms (Kawashima et al., 2005) and particularly the gene expression level of mitochondrial SERAT2;2 is high in Arabidopsis green leaf (Watanabe et al., 2015; Watanabe and Hoefgen, 2017).

### Comparison of Metabolic Changes Between *serat* Mutants and Mutants in Serine Biosynthesis

Metabolite profiles of a variety of mutants in photorespiratory and phosphorylated pathways of serine biosynthesis have been reported (Collakova et al., 2008; Cascales-Miñana et al., 2013; Kuhn et al., 2013; Toujani et al., 2013). The *shm1* mutant – deficient in the photorespiratory pathway, displayed a chlorotic phenotype with a dramatic increase in the levels of most amino acids including glycine and serine whilst alanine, aspartate and glutamate were decreased under ambient CO_2_ conditions, probably as they can donate their amino groups to photorespiratory glyoxylate instead of serine (Liepman and Olsen, 2003; Collakova et al., 2008; Kuhn et al., 2013). In contrast, knockout mutants, artificial microRNA lines or overexpression lines of the genes in the photorespiratory pathway only showed moderate metabolic responses but with an unexpected complexity (Cascales-Miñana et al., 2013; Toujani et al., 2013). The metabolic adaptations occurred independently of the serine levels, their genetic background or tissues (leaves and roots). Such a complexity of metabolite changes was also observed in the *serat* mutants in this study (**Figure 5**). These results suggest that the metabolic adaptations are to a certain extent flexible enough to compensate even for significant changes in the pathway. The fact that many of the metabolite levels could be maintained even in the growth-retarded *serat* mutants (**Figure 5**) suggested that there is a metabolic control system to maintain their levels during changes in metabolic flux at the expense of growth (Sieh et al., 2013). To understand the mechanism of metabolic control, a detailed metabolic flux analysis and metabolite profiling of subcellular fractions of *serat* mutants and serine biosynthetic mutants, maybe even grown under different growth conditions, should be employed in future studies.

### Interconnection Among Serine, Tryptophan and Indolic GSL Metabolism

Serine is also a substrate for the biosynthesis of tryptophan (Tzin and Galili, 2010), which is furthermore a precursor of the synthesis pathways of indolic GSLs and of the phytohormone indole acetic acid (Glawischnig et al., 2000; Gigolashvili et al., 2007; Vernoux et al., 2010). Recent studies provided some evidence for the link between the phosphorylated pathway of serine biosynthesis and tryptophan biosynthesis in plastids (Benstein et al., 2013); the gene expression of the enzymes in the phosphorylated pathway, phosphoglycerate dehydrogenase (PGDH1) and phosphoserine aminotransferase (PSAT1), are regulated by MYB51 and MYB34, which are also activators of tryptophan and indolic GSL biosynthesis. On the other hand, a recent *in vitro* study of PGDH proteins of Arabidopsis revealed that PGDH1 activity was inhibited by serine, but activated by cysteine, homocysteine, methionine, homoserine, valine, and alanine (Okamura and Hirai, 2017), indicating that sulfur metabolism also regulates the phosphorylated pathway. Furthermore, MYB51 and MYB34 are regulated by sulfur status (Frerigmann and Gigolashvili, 2014; Bielecka et al., 2015) and sulfur deficiency induced (SDI) proteins acting as major repressors controlling GSL biosynthesis in Arabidopsis (Aarabi et al., 2016). These findings suggest that three metabolic areas cross-regulate each other to maintain serine synthesis in plastids for tryptophan- and sulfur-metabolism and their respective downstream metabolic pathways. In this study, contents of serine, tryptophan, and total indolic GSLs were not significantly altered in any of the *serat* mutants (**Figures 3–5**). The adjustments of PGDH activity and the expressions of the *MYB*s by the reduced levels of sulfur metabolites in the *serat* mutants might play a role for keeping serine synthesis constant in plastid.

### Changes in GSL Compositions in *serat* Mutants

The changes of GSL compositions with different chain lengths in the three growth-retarded quadruple mutants (**Figure 4**) were like those in leaves of Arabidopsis grown under low sulfur conditions although total amounts of aliphatic and indolic GSLs were additionally decreased in the sulfur starved plants (Aarabi et al., 2016; Supplementary Figure 1A). This suggests that the compositional changes observed in the *serat* mutants are part of the sulfur-deficiency stress response. Different GSLs are assumed to have different roles for resistance responses to herbivores and pathogens (Kroymann et al., 2003; Clay et al., 2009), but the impact of the compositional changes was not studied in detail. Several studies of the enzymes such as MAMs (methylthioalkylmalate synthase) and CYP79Fs (Chen et al., 2003; Textor et al., 2007) in the indolic GSL pathway, which have different substrate specificities for short- and long-chain intermediates of aliphatic GSLs, suggested that there are specific regulations of GSL distributions in different tissues. Short-chain GSLs are produced and stored mainly in rosette leaves whilst long-chain aliphatic GSLs are produced mainly in roots as a source while leaves act as a sink (reviewed in Andersen et al., 2013; Moussaieff et al., 2013; Andersen and Halkier, 2014; Jørgensen et al., 2015; Supplementary Figure 1B). The change in the ratio of long- and short-chain GSLs in leaves of the quadruple mutants (**Figure 4**) could be caused by metabolite changes in not only leaves, but also in roots. More detailed analyses with both leaf and root tissues will be required in order to comprehensively understand sulfur-deficiency stress responses including long-distance transport of sulfur metabolites between source and sink tissues.

### Decrease of Sulfate Content in *serat* Mutants

A decrease of the sulfate content in the *serat* quadruple mutants (**Figure 3**) was observed under soil growth conditions of this study as well as in the mutants grown under the agar-plate conditions (Watanabe et al., 2008b, 2010), suggesting changes in sulfate transporter activity such as sulfate uptake in root or sulfate transport between tissues. It was reported that OAS and thiols (cysteine and GSH) positively and negatively regulate the gene expressions or activities of sulfate transporters, respectively (Brunold and Schmidt, 1978; Lappartient et al., 1999; Westerman et al., 2001). In sulfur-starved plants, OAS is increased, but thiols are decreased (Nikiforova et al., 2005; Hoefgen and Nikiforova, 2008), both of which activates the sulfate transporters whilst in the *serat* mutants, the contents of both, OAS and thiols, are decreased. Considering these points, the OAS reduction rather than the reduction of thiols might regulate the sulfate transporters in the *serat* mutants. However, the reduction of the levels of sulfate contents in the *serat* mutants were not always correlated with the contents of OAS and thiols (**Figure 3**; Watanabe et al., 2008b, 2010). The detailed analysis of the effects of OAS and thiols on the sulfate transporters including the balance and the levels of these metabolites in each subcellular localization or tissue might help to reveal the respective regulatory mechanisms.

## Conclusion

Serine acetyltransferase acts as the only interconnection between glycine/serine and sulfur metabolism. Complex regulatory patterns determine the interplay between sulfur metabolism and serine metabolism which are not only dependent on available enzyme activities but are also dependent on growth conditions, environmental and biotic stresses, and maybe developmental stages which determine whether a regulatory crosstalk between the metabolic areas can be observed. Further, complex links to tryptophan/auxin metabolism and GSL metabolism can be envisaged. The signals and the regulation of these complex responses is far from being understood. As a result of this investigation it is further advisable to investigate this system under various developmental and external conditions in order to ultimately unravel its full complexity.

## Materials and Methods

### Plant Material

Plants were cultured on soil (type GS-90 Einheitserde; Gebrüder Patzer) in a growth chamber under long day (16-h day, 140–160 μmol m^-2^ s^-1^, 20°C; 8-h night, 16°C). We harvested 25-day-old plants 4 h after the onset of light. Samples were immediately frozen in liquid nitrogen and stored at -80°C until further use. Three biological replicates were used for all analyses described in this study.

### SERAT Enzymatic Activity Assays

Serine acetyltransferase enzymatic activity assays were performed as described previously (Watanabe et al., 2008b). Frozen ground material (20 mg) was homogenized in 100 μL of extraction buffer containing 250 mM potassium phosphate, pH 8.0, 0.5 mM EDTA, and 10 mM 2-mercaptoethanol. The soluble protein amount was measured with the Bio-Rad Bradford reagent (Bio-Rad Laboratories) according to the manufacturer’s instructions. The enzymatic activity of SERAT was determined in reaction mixtures (100 μL) containing 50 mM Tris–HCl, pH 8.0, 1 mM acetyl-CoA, 10 mM serine. The reaction was performed at 30°C for 15 min and terminated by the addition of 10 μL of 7.5% (w/v) trichloroacetic acid. SERAT activity was determined for the production of OAS, which was derivatized with *O*-phthalaldehyde using HPLC (see section “Determination of Amino Acid Contents”).

### Extraction for Metabolite Profiles

Metabolites were extracted from frozen ground material (50 mg) in 300 μL of methanol using a mixer mill (Retsch, Germany) with a zirconia bead for 1 min at 25 Hz. Water-soluble metabolites were separated by adding 200 μL of chloroform and 400 μL of water to the extract. Subsequently, the polar phase was transferred into clean Eppendorf tubes. Samples were dried by vacuum centrifugation for 3 h and stored at -80°C until further use.

### Determination of Ion Contents

The evaporated polar fraction (100 μL) was dissolved in de-ionized water (550 μL) and analyzed by Dionex ICS-3000 system with a KOH gradient for anion analysis and with a methanesulfonic acid gradient for cation analysis according to the manufacturer’s instructions (Dionex, Idstein, Germany).

### Determination of Thiol Contents

The evaporated polar fraction (100 μL) was dissolved in 20 μL of 0.1 M HCl. A mixture of 20 μL of polar extract and 40 μL of 25 μM *N-*acetyl-Cys as the internal standard was incubated with 4 μL of 25 mM tris(2-carboxyethyl)phosphine and 10 μL of 1.58 M *N-*ethylmorpholine for 20 min at 37°C. The reduced thiols were labeled with 4 μL of 25 mM monobromobimane in acetonitrile for 20 min at 37°C in the dark. The labeling reaction was terminated by the addition of 10 μL of acetic acid and the resulting solution was then subjected to HPLC analysis. HPLC was carried out as described previously (Anderson, 1985; Fahey and Newton, 1987; Saito et al., 1994).

### Determination of Amino Acid Contents

The evaporated polar fraction (100 μL) was dissolved in 30 μL of 0.1 M HCl and subjected to HPLC analysis using a HyperClone C18 (ODS) column (Phenomenex, Aschaffenburg, Germany). Amino acids were measured by pre-column online derivatization with *O*-phthalaldehyde in combination with fluorescence detection (Lindroth and Mopper, 1979; Kim et al., 1997).

### Measurement of Primary Metabolites by Gas Chromatography/Time of Flight-Mass Spectrometer (GC/TOF-MS)

Metabolite profiling by GC/TOF-MS was performed as described previously (Lisec et al., 2006; Erban et al., 2007). The evaporated polar fraction (100 μL) was derivatized by methoxyamination and subsequent trimethylsilylation. Samples were analyzed using GC/TOF-MS (ChromaTOF software, Pegasus driver 1.61; LECO). The chromatograms and mass spectra were evaluated using TagFinder software (Luedemann et al., 2008) and Xcalibur 2.1 software (Thermo Fisher Scientific, Waltham, MA, United States). Metabolite identification was supervised using the mass spectral and retention index collection of the Golm Metabolome Database (Kopka et al., 2005; Hummel et al., 2010). Peak areas of the mass fragments were normalized on the basis of the fresh weight of the sample and the added amount of an internal standard ([^13^C_6_] sorbitol).

### Measurement of Secondary Metabolites by Liquid Chromatography/ElectroSpray Ionization-Mass Spectrometry (LC/ESI-MS)

The evaporated polar fraction (100 μL) was dissolved in 80% methanol (150 μL) containing isovitexin (5 μg mL^-1^) as the internal standard and measured by LC/ESI-MS as described previously (Tohge and Fernie, 2010). All data were processed using Xcalibur 2.1 software (Thermo Fisher Scientific, Waltham, MA, United States).

## Author Contributions

MW: experimental design, plant growth and harvesting, metabolite analysis, activity assay, prepared all figures and tables, and wrote the first draft. TT: experimental design, metabolite analysis, data interpretation, and revising the manuscript. AF and RH: experimental design, data interpretation, and revising the manuscript.

## Conflict of Interest Statement

The authors declare that the research was conducted in the absence of any commercial or financial relationships that could be construed as a potential conflict of interest.
