# Supplementary figures and images for: The Effect of Single and Multiple SERAT Mutants on Serine and Sulfur Metabolism
Source: Front Plant Sci. 2018 May 28;9:702. doi: 10.3389/fpls.2018.00702 (PMC5985473; doi:10.3389/fpls.2018.00702)

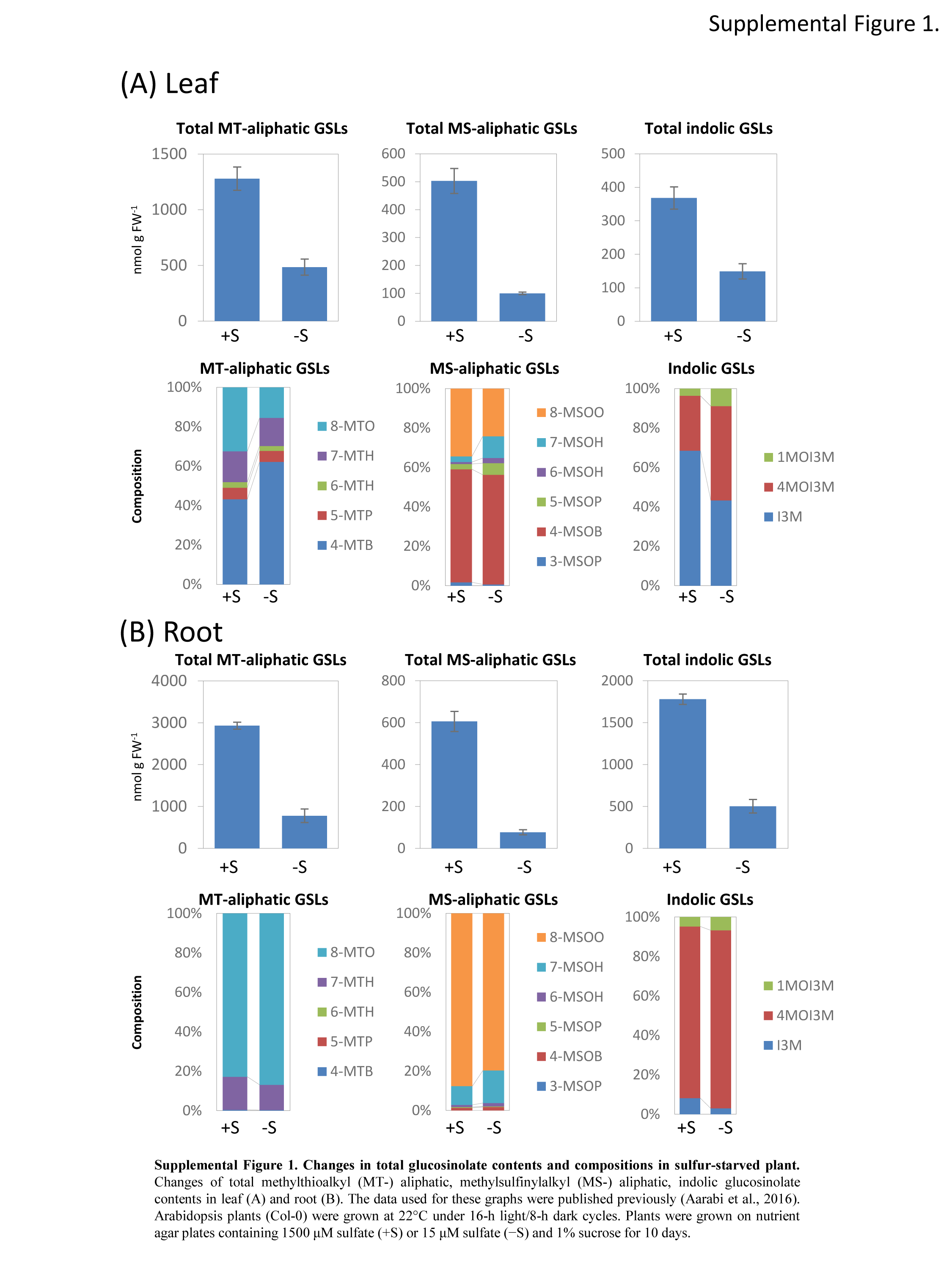

Supplement: Supplementary file 1 [file Image_1.TIF]
